# The Use of Horse Hair in Surgery

**Published:** 1895-03

**Authors:** C. M. Daniels

**Affiliations:** Chief Surgeon W. N. Y. & P. R’y., Buffalo, N. Y.


### The Use of Horse Hair in Surgery.

BY C. M. DANIELS, M. D.
Chief Surgeon W. N. Y. & P. R'y., Buffalo, N. Y.

In presenting this subject I lay no claim to originality in the use of horse hair in surgical practice, as it was suggested to me ten years ago; but having used it extensively during the period mentioned, and not having seen the subject referred to but once in surgical literature, I venture to present this paper which will certainly be commended for its brevity.

For many years, surgeons have been faithfully searching for the ideal suture, one that would be non-irritating, small in caliber, easily adjusted and afford the closest approximation of the skin layers, and this with special reference to wounds of the face or exposed parts where the avoidance of scars, more or less unsightly, is a great desideratum.

Catgut, silk and silkworm gut have long been used, but in the first we have a rapidity of softening and relaxation that will not infrequently result in a gaping wound. In the second, the resulting "stitch marks" which are frequently long in disappearing, added to the occasional irritation, and in the silkworm gut the rigidity of the suture itself makes each undesirable in superficial wounds, and all require dressings or coverings held by bandages, which are both disagreeable and cumbersome, especially when the patient is able to be about or upon the street. I speak with special reference to those wounds, which in themselves, are not serious enough to confine the patient, this being the class of cases where I feel free to recommend horse hair sutures, and upon which I note the following points:

1. Instead of a large or medium sized needle as required with even small strands of catgut, silk or silkworm gut, with the horse hair a small eye needle, curved or straight, according to the preference of the surgeon, can be used, and thus minimize the size of each puncture of the skin.

2. Horse hair, when prepared as I will suggest later, has a firmness that will give support to the skin, and very materially aid in keeping in approximation the edges of the same, and more nearly resembles silkworm gut in its action in the tissue.

3. Where wounds are both lacerated and contused, with the fine needle, we can pick up many small but valuable parts which may be saved by gentle adjustment, and I will mention here that success, in any case, depends upon the care used in the introduction on one side, and exit on the other, of the needle near the margin of the cut or torn surface, which should always be at right angles to the incision or skin margin, so that when the tie is made the strand will be of the same length upon the under as upon the upper side, and at a little to one side of the wound line.

4. Horse hair sutures can be placed much closer together than other suture material, with less danger of strangulation of the skin margins, this in itself better assuring perfection of adjustment and more satisfactory results, and a "reef" or "granny" knot only, is required, being smaller than the ordinary surgical tie.

5. Horse hair *will not* bear any great amount of tension, and could hardly be recommended for use in the flaps after a major amputation, except to place between other sutures for the purpose of more carefully approximating the skin margins.

6. After the introduction of horse hair, the skin being accurately adjusted and operation having been performed under the aseptic surgical procedures of to-day, except in excessive injuries, no dressing is required other than carefully drying the parts and brushing over with a layer of aseptic flexible collodion, which, as it dries, will aid the horse hair in holding the skin in position.

With special reference to facial wounds, the field for the fastidious surgeon is here a great one, as horse hair at almost any hue of fast colors can be obtained, and he can place almost invisible stitches, from the color of the skin of the albino to that of the darkest African.

And now a word relative to the preparation of horse hair for surgical purposes: The convenience of the stable places it in every surgeon's hands, and the hair needs only a thorough washing with soap and water, then for two or three days in 1 to

1,000 hydrarg. bichlo. solution, and transferred to 95 per cent. alcohol until used. I believe that when this material is more generally used and appreciated, that surgical supply houses will place it upon the market, and I have already made such a suggestion to one of them, and without hope of reward.—*Journal Am. Med. Assn.*

